# Psychological wellbeing and physical activity in children and adolescents with inflammatory bowel disease compared to healthy controls

**DOI:** 10.1186/s12876-017-0721-7

**Published:** 2017-12-12

**Authors:** Laura Mählmann, Markus Gerber, Raoul I. Furlano, Corinne Legeret, Nadeem Kalak, Edith Holsboer-Trachsler, Serge Brand

**Affiliations:** 1 Psychiatric Clinics of the University of Basel, Centre for Affective, Stress and Sleep Disorders, University of Basel, Wilhelm Klein-Strasse 27, Ch-4012 Basel, Switzerland; 2 Pediatric Gastroenterology & Nutrition, University Children’s Hospital Basel, Basel, Switzerland; 3 Department of Sport, Exercise and Health, Sport Science Section, University of Basel, Basel, Switzerland; 4 United Nations University - Maastricht Economic and Social Research Institute on Innovation and Technology (UNU-MERIT), Maastricht University, Maastricht, The Netherlands; 5 Substance Abuse Prevention Research Center; Sleep Disorders Research Center, Psychiatry Department, Kermanshah University of Medical Sciences, Kermanshah, Iran

**Keywords:** Inflammatory bowel diseases, Pediatrics, Anthropometric dimensions, Psychological wellbeing, Physical activity, Blood values, Healthy controls

## Abstract

**Background:**

Children and adolescents with inflammatory bowel disease (IBD) report impairments in daily activities, social interactions and coping. Findings regarding psychological functioning are inconsistent, while limited information is available on objectively assessed physical activity (PA). The aims of the present study were therefore to compare anthropometric dimensions, blood values, psychological functioning and PA of children and adolescents with IBD with healthy controls.

**Methods:**

Forty-seven children and adolescents took part in the study. Of these, 23 were diagnosed with IBD (mean age: 13.88 years, 44% females). The IBD group was divided into a medically well adjusted “remission-group” (*n* = 14; IBD-RE) and a group with an “active state” of disease (*n* = 8; IBD-AD). Healthy controls (*n* = 24; HC) were age- and gender-matched. Participants’ anthropometric data, blood values and objective PA were assessed. Further, participants completed questionnaires covering socio-demographic data and psychological functioning.

**Results:**

Participants with IBD-AD showed higher erythrocyte sedimentation rate (ESR), C-reactive protein (CRP) values, haemoglobin, and leukocyte values. IBD-AD had poorer psychological functioning and lower PA (average steps per day) compared to IBD-RE and HC. No mean differences were found between IBD-RE and HC.

**Conclusions:**

The pattern of results suggests that effective medical treatment of IBD in children and adolescents is associated with favorable physiological parameters, psychological dimensions and PA. Psychological counselling of children and adolescents in an active state of IBD seem to be advised in addition to standard treatment schedules.

**Trial registration:**

NCT NCT02264275; Registered 8 October 2014.

## Background

Inflammatory Bowel Disease (IBD) is a chronic, debilitating illness characterized by cycles of disease activity and quiescence. IBD is subdivided into Crohn’s disease (CD), ulcerative colitis (UC) and atypical phenotypes as described by the Porto criteria (PIBD: Pediatric IBD) [1]. Although the ultimate aetiology of IBD remains unclear, three hypotheses are advanced: (1) A dysregulated inflammation emerges due to interactions between the gut luminal content (intestinal microflora) and the mucosa, especially in genetically predisposed hosts [2]. (2) Genetic factors seem to contribute slightly to the disease pathogenesis. (3) Microbial and environmental dimensions have been identified as possible contributing factors [3].

Worldwide it is estimated that 25% of all patients suffering from IBD are children and adolescents, with increasing incidence rates [4]. In children with IBD stunting of physical growth and delay in pubertal development are observed [5]. Furthermore, patients with active IBD are known to report increased erythrocyte-sedimentation rates (ESR) due to inflammation, decreased hemoglobin due to chronic blood loss in the gut, decreased albumin due to leaking gut at reduced intake and increased infection parameters such as leukocytes and C-reactive protein (CRP), when compared to healthy controls. Once the diagnosis is confirmed, the goals of medical therapy are to relieve symptoms, restore growth and bone health, normalize quality of life and psychosocial functioning, prevent complications and minimize the adverse effects of medications [6]. While the efficacy of anti-inflammatory pharmaceuticals are not 100% and the side effects might be severe, some young IBD patients might be in a symptom-free remission, while others are still in an active disease state. Accordingly, based on the regularly assessed PUCAI (Pediatric ulcerative colitis activity index) scores and pediatric Crohn’s disease activity index (CDAI), participants in the present study were split into those with medically optimized treatment (IBD in remission, PUCAI <10; CDAI <150) the IBD – RE group, and those with not yet medically optimized treatment (active disease state), the IBD – AD group. As a result, our first hypothesis was that disease severity and the accompanying symptoms would be negatively associated with the physical development of children and adolescents with IBD. Specifically, we expected smaller and lighter body shapes and elevated inflammation markers in participants of the IBD-AD group, compared to IBD-RE and healthy controls.

In recent years, a plethora of studies have suggested that the prevalence of reduced health-related quality of life (HRQOL) and psychiatric disorders are significantly greater in young people with IBD than healthy controls [7–9]. More specifically, adolescents with IBD had higher levels of internalizing disorders such as anxiety and depression [7, 9], due to several factors such as unpredictable, unpleasant, and embarrassing symptoms, complex, demanding treatment regimens, treatment-related side effects, the ever-looming threat of exacerbations of the disease, and the need for surgical procedures in severe cases [10]. The rate of depression may be as high as 25%, and spreads among a broad variety of psychological and social difficulties. However, as reviewed by Ross et al. [8], the evidence these conclusions are based on, were inconsistent, included few paediatric samples, and used coarse-grained psychological instruments. As a result, recognition of the importance of mental wellbeing in young IBD patients is lacking in medical practice. If these psychological conditions are left untreated, mental disorders linked to more severe IBD symptoms might emerge, as well as more frequent IBD flares, higher hospitalization rates, increased health seeking behaviour and lower compliance with treatment [11, 12]. To counter this, in the present study, validated and reliable self-rating instruments were used to assess dimensions such as psychological wellbeing, social support and peer relationships. Consequently, the second aim of the present study was to expand upon existing research by assessing children’s and adolescent’s psychological functioning with self-rating questionnaires, investigating specific aspects of psychological functioning, along with a depression screener.

As regards physical activity and sports participation, research showed that children and adolescents with IBD participated less in sports activities and were less fit than their counterparts without IBD [13, 14]. In the specific case of paediatric IBD, reduced physical exertion during puberty might lead to impaired bone growth, strength and density, resulting in an increased risk for osteoporosis in the long term [10]. However, a large number of studies have proven the beneficial effect of PA for a diversity of health conditions: First, PA mitigates depressive symptoms while bolstering sleep [15] and cardiovascular fitness [16]. Second, sports participation (via clubs and sports associations) has the potential to increase and deepen children’s and adolescents’ social skills. So far, research on PA in children and adolescents with IBD has largely been based on rough estimates. Therefore, the third aim of the present study was to assess PA behavior in children and adolescents with IBD, both subjectively using an internationally established questionnaire, and objectively with three methods: First, to investigate participants’ fitness, we used the Six-Minute-Walking-Test; second, for strength assessments, we applied a hydraulic grip strength test, and third, to generate long-term PA reports, a FitbitFlex® accelerometer was applied.

Taking into account the state-of-the-art regarding physical development, psychological functioning and physical activity among children and adolescents with IBD, the following three hypotheses were formulated: First, following others [5, 17], we expected impaired anthropometric development among children and adolescents suffering from IBD and increased inflammatory blood markers, and particularly among those in an active state of the disease. Second, on the basis of previous research [7–9], we expected poorer psychological functioning in children and adolescents with IBD, than in their healthy counterparts, and again, particularly among those in an active state of the disease. Third, previous findings [18] led us to expect lower levels of PA (both subjective and objective) in children and adolescents with IBD than in their healthy counterparts.

## Methods

### Procedure

All participants were invited to the hospital to measure height, weight, waist circumference (measured 4 cm above the navel using a standard anthropometry tape) compared to reference values established by Taylor et al. [19] and BMI (kg/m^2^), which was compared to the WHO international growth references [20]. Following these anthropometric measurements, participants’ blood values were collected and physical performance was examined; they also filled out a 30 min questionnaire with questions on psychological functioning and PA behaviour. All data were collected by two trained research assistants and supervised by a medical doctor.

The present study was approved by the local ethical committee (EKNZ: 2014:220), and was conducted in accordance to the ethical principles laid down in Declaration of Helsinki (Trial registration number: NCT02264275).

### Sample

A total of 31 eligible children and adolescents with IBD were approached between April and November 2015 via the University Children’s Hospital Basel (UKBB, Basel, Switzerland). Of these, 29 (93.5%) agreed to participate in the study, though five subsequently withdrew from the study because of time constraints, and three withdraw due to acute illness (flu). The final sample consisted of 23 children and adolescents with diagnosed IBD (mean age: 13.88 years, SD = 3.11, 10 females (43.5%), 7 UC, 12 CD and 3 UD). According to the regularly assessed PUCAI (Paediatric ulcerative colitis activity index) scores and Paediatric Crohn’s disease activity index (clinical scores), eight participants were in an active disease state and 14 in remission at the time of the study [11]. Inclusion criteria were as follows: (1) aged 6 to 20 years; (2) clinically and histologically confirmed diagnosis of IBD; (3) willing and able to take part in the study; (4) able to communicate and to complete questionnaires in German; (5) written informed consent from both the children/adolescents and their parents (or a legal caregiver). Exclusion criteria were: (1) severe physical diseases of the locomotor apparatus; (2) psychiatric disorders such as psychotic disorders, severe affective disorders, eating disorders, mental retardation, autism spectrum disorder; (3) unable to communicate and to complete questionnaires in German; (4) among female adolescents: pregnancy, breastfeeding, or intention to get pregnant during the study period; (5) refusal to give written informed consent. In parallel, 24 age- and gender-matched controls (mean age: 12.38 years, SD = 3.24, females *n* = 15 (62.5%)) were recruited by word of mouth recommendations. Inclusion criteria and exclusion criteria for healthy controls were the same as for patients with IBD except that they had to be physically healthy as reported from both participants and parents and as known from medical records. All participants were informed about the study aims and the voluntary and confidential basis of their participation. Written informed consent was signed both by participants and by their legal guardians.

Characteristics of the participants subdivided into three groups of IBD-AD, IBD-RE and healthy control are represented in Table 1.

**Table 1:** Sample characteristics

	IBD-AD	IBD-RE	HC
	(*n* = 8)	(*n* = 14)	(*n* = 24)
Age in years	14.69 ± 3.25	13.23 ± 2.96	12.38 ± 3.24
Female	4 (50)	6 (42.9)	15 (62.5)
IBD			
*Ulcerative colitis*	3 (37.5)	4 (28.6)	
*Crohn’s disease*	5 (62.5)	7 (50)	
*Undefined colitis*		3 (21.4)	
Time since diagnosis (in years)	3.67 ± 2.81	4.1 ± 2.9	

Notes: *N* = 46

*IBD-AD* IBD in an active state of the disease, *IBD-RE* IBD in remission, *HC* Healthy control

### Tools

#### Laboratory assessment

Using the finger prick technique, biochemical parameters were assessed involving inflammatory indices such as albumin [g/dl], CRP [g/dl], hemoglobin [g/dl], ESR [mm/h], hematocrit [%], thrombocyte [g/dl] and leucocyte [g/dl] count.

#### Assessing psychological functioning

To assess psychological functioning, participants completed the KIDSCREEN 27 [21]. The questionnaire consists of 27 items covering five domains, physical wellbeing, psychological wellbeing, autonomy and relations with parents, social support and peers, and school environment. Answers were given on 5-point rating scales (1 = not at all, 5 = extremely/always). Higher mean scores reflect better functioning in a specific domain. Validity has been verified by Ravens-Sieberer et al. [21] (Cronbach’s α = .91).

To assess symptoms of depression, the Child Depression Screener (ChilD-S) was completed [22]. The ChilD-S is a self-report screening instrument for pre-pubertal in- and out-patients in paediatric care. It consists of 8 items assessing how participants have felt for the last 2 weeks (“I am happy, I am doing fine, I feel exhausted, I worry a lot, I feel sad, I get upset quickly, I am not in the mood for anything, I often think I did something wrong”). Participants were asked to select from four alternative responses reflecting different levels of depressive symptomatology. The cut off value for clinically considerable depression is ≥11, with higher values indicating more marked depressive symptoms. Validity has been verified by Frühe et al. [22] (Cronbach’s α = .78).

#### Assessing physical activity

##### Subjective assessment

The short form of the IPAQ (IPAQ-S) questionnaire was applied as an internationally approved estimator of level of PA and sedentary behaviour [23]. It provides a comparison of vigorous- and moderate-intensity PA, walking, total PA and time spent sitting on weekdays over the past seven days. PA data are reported in hours and/or minutes per day and days per week. Where minutes of intense activity exceeded 180 per day, a cut-off of 180 min per day was applied as suggested by the guidelines of the IPAQ group. Data were transformed and summed using standardized IPAQ scoring protocols to indicate total metabolic equivalent minutes (MET-minutes) of PA per week. Total MET-minutes per week was calculated using the following formula [23]:

$$ {\displaystyle \begin{array}{l}\left[\mathrm{Walking}\ \mathrm{MET}\hbox{-} \mathrm{minutes}/\mathrm{week}={3.3}^{\ast }\ {\mathrm{walking}\  \mathrm{minutes}}^{\ast }\ \mathrm{walking}\  \mathrm{days}\right]\ \\ {}+\left[\mathrm{Moderate}\ \mathrm{MET}\hbox{-} \mathrm{minutes}/\mathrm{week}={4.0}^{\ast }\ \mathrm{moderate}\hbox{-} \mathrm{intensity}\ {\mathrm{activity}\  \mathrm{minutes}}^{\ast }\ \mathrm{moderate}\  \mathrm{days}\right]\ \\ {}+\left[\mathrm{Vigorous}\ \mathrm{MET}\hbox{-} \mathrm{minutes}/\mathrm{week}={8.0}^{\ast }\ \mathrm{vigorous}\hbox{-} \mathrm{intensity}\ {\mathrm{activity}\  \mathrm{minutes}}^{\ast }\ \mathrm{vigorous}\hbox{-} \mathrm{intensity}\  \mathrm{days}\right]=\mathrm{Total}\ \mathrm{PA}\ \mathrm{MET}\hbox{-} \mathrm{minutes}/\mathrm{week}.\end{array}} $$

Furthermore, self-reported fitness was assessed on a 5-point Likert scale. Validity of the instrument has been established by Hagstömer et al. [24] Sitting time was reported as the amount of time in hours and/or minutes participants usually spent sitting on a weekday during the past 7 days.

##### Objective assessments

*Grip force.* Maximum isometric grip force of the dominant hand was assessed using the hydraulic hand dynamometer. Participants made three attempts. Mean outcomes were compared to reference data [25].

*Functional capacity.* The 6-min walking test (6 MWT), a self-paced, submaximal exercise test, was employed [26]; the test is designed to assess functional exercise capacity in patients with chronic diseases. The 6 MWT is well-standardized and is increasingly being utilized in pediatric populations with chronic diseases [27]. Walking distance is accepted as the main outcome measure of the 6 MWT. Heart rate (HR) is continuously recorded during exercise using an elastic chest strap with heart rate sensor.

*Daily PA.* The Fitbit-Flex® (Fitbit Inc., San Francisco, CA, USA), a small and light wristband accelerometer which uses three-dimensional motion sensing technology to measure movement 24 h a day, was applied for a continuous assessment of 5 days (2 weekend days and 3 weekdays). It has a simple display of five LED lights which indicate the number of steps taken in a day. Fitbit data were recorded using the 1-min epoch setting and downloaded from the user website via the device’s USB docking port in a raw data format. Validation studies have been conducted with children and adolescents [28].

### Statistical analyses

Since normality was violated according to Shapiro-Wilk test, the groups IBD-AD, IBD-RE and HC were compared using the Kruskal-Wallis test with these diagnostic clusters as independent variable and anthropometric measures, psychological assessments and PA data as dependent variable. Due to heterogeneity of age distribution, Spearman’s correlations were performed to determine whether significant correlations would be found beyond age and other dimensions. Accordingly, statistical calculations of anthropometric data, grip strength and 6 MWT were controlled for age. Post-hoc tests after Whitney-U for *p*-values were performed to examine differences between the three groups. The nominal level of statistical significance was set at alpha <.05. Further, effect sizes were reported as partial eta-squared [*η*_*p*_^*2*^]) and considered as follows: small (s) = .01 ≤ *η*_*p*_^*2*^ ≤ .059, medium (m) = .06 ≤ *η*_*p*_ ≤ .139, or large (l) = *η*_*p*_ ≥ .14) [29]. All statistics were performed with SPSS® 24.0 (IBM Corporation, Armonk NY, USA) for Windows®.

## Results

All descriptive and inferential statistical information is reported in the Tables and not repeated again in the written text. All Tables compare the three groups of IBD-AD, IBD-RE and HC.

### Anthropometric and laboratory findings

As shown in Table 2, there was a statistically significant mean difference in thrombocytes (*p* = .003). According to Mann-Whitney-U Post-hoc test, differences between IBD-AD and HC were significant (*p* = .003). Further, there were no statistically significant mean differences between the three groups in the following dimensions: waist circumference, waist circumference difference from norm, height, weight to height percentage, weight, BMI, BMI z-score, percentile BMI vs age, z-score height vs age, percentile height vs age and the blood values albumin [g/l], CRP [mg/l], hemoglobin [g/l], ESR [mm/h, hematocrit [%] and leukocytes [×10^9/l]. Investigating effect sizes, the IBD-AD group scored moderately lower on BMI z-score, percentile BMI vs age, z-score height vs age and percentile height vs age.

**Table 2:** Anthropometrics and Blood values

	IBD-AD	IBD-RE	HC	Statistical Analysis			
	(*N* = 8)	(*N* = 14)	(*N* = 24)				
	*M ± SD*	*M ± SD*	*M ± SD*	*H*	*p*	ηp2	Interpretation of effect size
Waist circumference (cm)	67.63 ± 9.04	65.65 ± 7.17	64.48 ± 8.29	0.384	0.823	0.01	s
Difference from norm	−9.81 ± 6.15	−8.68 ± 4.95	−8.82 ± 6.0	0.521	0.783	0	s
Height (cm)	153.48 ± 17.53	152.69 ± 12.02	152.26 ± 18.0	0.212	0.903	0	s
Weight to height (%)	44.08 ± 3.07	42.94 ± 2.13	42.77 ± 3.49	1.108	0.585	0.02	s
Weight (kg)	45.28 ± 15.85	42.79 ± 12.83	44.37 ± 15.75	0.215	0.899	0	s
BMI	18.6 ± 3.37	17.91 ± 2.78	18.36 ± 3.16	0.074	0.965	0.12	m
BMI z-score	−0.588 ± 0.79	−0.34 ± 0.95	−0.19 ± 0.87	1.368	0.511	0.1	m
Percentile BMI vs Age	31.98 ± 24.31	39.82 ± 26.23	43.89 ± 26.43	1.386	0.516	0.1	m
z-score Height vs Age	−0.911 ± 1.73	0.02 ± 0.86	0.19 ± 1.01	2.234	0.334	0.1	m
Percentile Height vs Age	32.04 ± 32.99	52.91 ± 25.71	54.83 ± 31.53	2.236	0.333	0.07	m
Albumin [g/l]	23 ± 21.94	19.32 ± 19.20	20.79 ± 18.76	0.767	0.686	0	s
CRP [mg/l]	3.02 ± 4.56	3.28 ± 5.69	0.71 ± 0.85	4.159	0.125	0.13	m
Hemoglobin [g/l]	139.25 ± 8.62	134.92 ± 16.26	135.86 ± 12.33	1.423	0.493	0.01	s
ESR [mm/h]	4.83 ± 0.36	4.87 ± 0.54	4.88 ± 0.36	0.281	0.874	0	s
Hematocrit [%]	39.4 ± 2.11	38.09 ± 3.99	38.75 ± 3.17	0.359	0.837	0.02	s
Thrombocyts [×10^9/l]	323.25 ± 55.61	302.58 ± 56.15	271.55 ± 46.54	11.211	0.003	0.13	m
Leukocyten [×10^9/l]	10.16 ± 2.01	9.28 ± 2.83	8.25 ± 1.29	4.088	0.13	0.11	m

Notes: *N* = 46, degrees of freedom always = 2, 41, *p* < .05 statistically significant; effect sizes: small (s) = .01 > ηp2 < .059, medium (m) = .06 > ηp < .139, or large (l) = ηp ≥ .14

*IBD-AD* IBD in an active state of the disease, *IBD-RE* IBD in remission, *HC* Healthy Control, Waist circumference norm references is Taylor et al. [19], *BMI* Body Mass Index; *CRP* C-reactive protein, *ESR* Erythrocyte Sedimentation Rate

Further, Table 2 shows higher C-reactive protein (CRP) values, hemoglobin values, and leukocyte values in the group of IBD-AD vs IBD-RE and HC. More specifically, six (75%) IBD-AD patients had low albumin levels, as did eight (61.54%) IBD-RE patients and 12 (50%) HC (reference 35 – 53 g/L). CRP was high in one (12.5%) IBD-AD and one (7.1%) IBD-RE patient (reference <10.0 mg/L). Two patients (25%) in the IBD-AD group, two IBD-RE (14.2%) and one control (4.2%) were anemic with mean hemoglobin scores of 101.5 g/L, 114 g/L and 100 g/L, respectively (reference 120 – 160 g/L).

Assessing the erythrocyte sedimentation rate (ERS) one IBD-AD (12.5%) was slightly above reference, as well as four (28.6%) IBD-RE and six (25%) HC (reference 4.1 – 5.1 × 10 ^12/L). One IBD-RE (7.1%) patient had results below reference.

Regarding hematocrit outcomes, two (25%) IBD-AD, four (28.6%) IBD-RE (mean 34.27%) and three (12.5%) HC had values below reference (36-46%). Thrombocytosis was present in two (25%) IBD-AD patients (reference 150 – 450 × 10^9/l). Leukocyte counts were high in two (25%) IBD-AD, three (21.4%) IBD-RE and one (4.2%) HC compared to reference (4.5 – 11 × 10^9/l).

### Psychological functioning

As Table 3 shows, there were no statistically significant mean differences between the three groups (psychological wellbeing physical wellbeing, autonomy and parent relations, social support and peers, school experience). Descriptively, IBD-AD had lower scores on the psychological wellbeing (PWB) dimension of the KIDSCREEN-27, while the other two groups did not differ from each other. The school dimension (SCH) showed a medium effect of .063, reflecting lower scores in the IBD-AD group.

**Table 3:** Psychological functioning and depression

	IBD-AD	IBD-RE	HC	Statistical Analysis			
	(*N* = 8)	(*N* = 14)	(*N* = 24)				
	*M ± SD*	*M ± SD*	*M ± SD*	*H*	*p*	ηp2	Interpretation of effect sizes
Kidsscreen-27:							
Physical Wellbeing	17.71 ± 3.59	18.5 ± 3.61	19.38 ± 3.16	3.740	0.155	0.04	s
Psychological Wellbeing	28.14 ± 5.61	31.86 ± 2.28	31.46 ± 2.41	3.258	0.197	0.16	l
Parent & Autonomy	31.14 ± 3.53	31.57 ± 3.17	31.479 ± 2.95	0.238	0.89	0	s
Peers & Social Support	16.71 ± 2.43	17.43 ± 2.38	17.29 ± 2.24	0.705	0.719	0.01	s
School Environment	15.57 ± 3.41	17.07 ± 1.86	17.29 ± 2.35	1.033	0.599	0.06	m
ChilD-S	6.75 ± 5.78	4.07 ± 3.29	3.75 ± 2.75	1.895	0.395	0.09	m

Notes: *N* = 46; degrees of freedom always = 2.41; p < .05 statistically significant; effect sizes: small (s) = .01 > ηp2 < .059. medium (m) = .06 > ηp < .139. or large (l) = ηp ≥ .14

*IBD-AD* IBD in an active state of the disease, *IBD-RE* IBD in remission, *HC* Healthy Control, *ChilD-S* Child Depression Screener

Results of the depression scale indicated a medium effect size of .092, with the highest depression scores in the IBD-AD group and no differences between IBD-RE and HC.

#### Physical activity

Table 4 shows that results on a subjective scale assessing the last 7 days, the IPAQ questionnaire did not indicate any statistically significant mean differences in self-reported PA between the three groups IBD-AD, IBD-RE and HC. On a descriptive level, the IBD-AD group reported more vigorous physical activity (medium ES of .088) than the IBD-RE and HC groups. However, self-estimated fitness was lowest in the IBD-AD group (ES = .109).

**Table 4:** Subjective and objective physical activity

	IBD-AD	IBD-RE	HC	Statistical Analysis			
	(*N* = 8)	(*N* = 14)	(*N* = 24)				
	*M ± SD*	*M ± SD*	*M ± SD*	*H*	*p*	ηp2	Interpretation of effect sizes
MET min vigorous	2610 ± 2745.99	2202.86 ± 1832.39	1378.33 ± 1023.34	2.124	0.355	0.09	m
MET min moderate	710 ± 789.5	557.14 ± 406.85	1198.33 ± 2032.29	0.594	0.752	0.04	s
MET min walking	315.56 ± 136.19	321.16 ± 390.79	397.79 ± 330.5	1.878	0.409	0.02	s
Total MET	3635.56 ± 2819.76	3081.16 ± 1994.34	2974.45 ± 2354.31	0.546	0.768	0.01	s
Sitting Weekend	382.5 ± 198.84	407.14 ± 193.25	351.33 ± 163.72	0.504	0.779	0.02	s
Sitting Weekday	411.25 ± 132.23	408.21 ± 143.41	384.38 ± 107.55	1.022	0.608	0.01	s
Fitness	2.25 ± 1.17	2.93 ± 0.92	3.08 ± 0.78	3.501	0.177	0.11	m
Heart Rate pre 6 MWT	94.63 ± 15.91	101.69 ± 13.19	94.26 ± 11.25	3.357	0.189	0.11	m
Heart Rate post 6 MWT	142.88 ± 46.41	161.92 ± 28.19	163.13 ± 28.9	0.623	0.74	0.05	s
Heart Rate increase	48.25 ± 45.99	60.23 ± 26.47	68.87 ± 30.35	0.801	0.679	0.07	m
6 MWT Distance	655.38 ± 135.69	719.08 ± 84.91	687.78 ± 88.02	1.247	0.544	0.07	m
Borg 1-10	4 ± 1.31	4.31 ± 2.72	3.74 ± 2.4	0.689	0.715	0.02	s
Grip strength (kg)	28.72 ± 11.61	24.54 ± 8.66	24.12 ± 9.64	0.877	0.65	0.02	s
Difference from norm	1.42 ± 9.34	−3.2 ± 8.25	−2.08 ± 7.36	0.837	0.663	0.03	s
Average daily steps	8049 ± 3614	10.689 ± 3089	12.473 ± 4248	5.923	0.049	0.18	l

Notes: *N* = 46. degrees of freedom always = 2.41; p < .05 statistically significant; effect sizes: small (s) = .01 > ηp2 < .059. medium (m) = .06 > ηp < .139. or large (l) = ηp ≥ .14

*IBD-AD* IBD in an active state of the disease, *IBD-RE* IBD in remission, *HC* Healthy Control, *MET* Metabolic Equivalent, *6 MWT* 6-min walking test

Mean grip strength did not differ statistically significantly between the three groups IBD-AD, IBD-RE and HC.

Even though statistically non-significant, the mean 6MWT results indicated descriptive differences, with IBD-AD patients achieving the shortest distance compared to IBD-RE and HC (medium ES = .070). The IBD-AD also had less increase in heart rate (medium ES = .073), but still rated the intensity of the test as high as the other two groups (small ES = .018).

For the objective step counts as measured by the FitbitFlex®, a statistical significant mean difference (*p* = .049) and a large effect size of .183 were observed; the average count was 8049 steps per day in the IBD-AD group, 10,689 steps per day in IBD-RE and 12,473 steps per day in the HC group. According to Mann-Whitney-U post-hoc tests, comparisons between groups were not significant.

## Discussion

The key findings of the present study were that children and adolescents with IBD-AD had poorer psychological functioning than children and adolescents with IBD-RE and HC. Furthermore, they had a lower functional capacity (6MWT) and engaged less in objectively assessed physical activity (average steps per day) compared to children and adolescents with IBD in remission or age-and gender-matched healthy controls. The present pattern of results adds to the current literature in an important way, showing that medically well-adjusted children and adolescents with IBD do not differ from healthy controls with regard to psychological functioning and objective and subjective PA.

Three hypotheses were tested and each of these is considered now in turn.

Our first hypothesis was that there would be anthropometric differences; specifically, we expected that participants with IBD were smaller, lighter and to have greater inflammation, especially among the more severe cases of IBD. Our data partly supported this hypothesis. The IBD-AD group had a moderately lower BMI z-score, BMI percentile, z-score height for age and percentile height for age when compared to both other groups. Therefore, the present data are in accordance with previous findings [14, 30]. In general, 10-40% of children with IBD are affected by growth failure [14, 30] and impaired nutritional status [31]. Underlying reasons are anorexia, malabsorption, intestinal inflammation and corticosteroid usage [32]. Given that the data available do not allow a deeper understanding of the pattern of results, we speculate that current treatment strategies lead to good disease control and less growth retardation [33].

With the first hypothesis, we also expected to detect indicators for inflammation and disease-related influences in the blood values of IBD patients. This hypothesis was partially supported; scores for the inflammatory markers ESR, CRP and leukocytes were higher among those with IBD-AD. Two patients with active IBD and two in remission were anemic, as well as one participant in the control group. In general, chronic anemia is prevalent and rapidly recurring in patients with IBD [34]. On the other hand, iron deficiency is a well-known phenomenon among many healthy female teenagers [35]. The small reported differences can be accounted to constant monitoring of blood values by the treating physician, who provides the patients with either oral or intravenous iron supplementation as soon as necessary. Looking at albumin values, it is not unusual to observe lower values in patients with IBD, as indicated by our findings. Patients with IBD might lose albumin due to increased gut permeability, even during symptom-free episodes [36]. However, we were unable to explain the low albumin values found in half of the healthy control participants. Common underlying reasons are undernourishment [37], decreased production due to liver disease [38] and increased excretion due to kidney problems [39], factors, which however could be ruled out by the supervising health professionals in the present study. Finally, the increased average hemoglobin among IBD-AD in comparison to HC was very surprising. The only possible explanation might be a blood clotting during blood sample taking since the hematocrit is elevated as well.

With the second hypothesis, we expected poorer psychological functioning in children and adolescents with IBD-AD compared to IBD-RE and healthy controls, and data did partially support this. Children and adolescents with increased disease activity had poorer psychological functioning in the area of psychological wellbeing. On the flipside, no other dimension of psychological functioning (physical functioning, autonomy and parent relations, social support and peers, and school experience) showed significant differences across the three groups, IBD-AD, IBD-RE and HC. Therefore, the present data do not match the study by Herzer et al. [40], showing a lower overall HRQOL among patients with IBD. Further, the present data do not accord with those studies reporting impaired physical functioning [41], impaired family functioning [40], limited participation in social activities [7], lower emotional functioning [42], and problematic school experience and performance.

Previous evidence can be explained by the association between increased disease activity and lower psychological functioning. One may claim that greater disease severity might be accompanied by an increasing presence of disruptive gastrointestinal symptoms and abdominal pain, which in turn need to be treated with more robust and invasive methods. Such increasing disease burden might result in psychological distress [43]. Two further hypotheses are the inflammation-depression hypothesis and the brain gut hypothesis. These two hypotheses claim that the increased production of pro-inflammatory cytokines (TNF alpha) is known to affect the brain both, directly and indirectly, thereby increasing symptoms of depression [44, 45]. Further, there is a bidirectional relationship, since psychological stress in turn increases the likelihood of inflammation, which again increases the occurrence of depressive symptoms [42]. The pro-inflammatory effect of experimental stress has been confirmed in human studies, and at the same time, inflammatory markers have been found to be raised in depressed patients [46]. To a wider extent, the inflammatory, unpredictable and disruptive nature of severe IBD, if not properly treated, could lead to an increase in internalizing symptoms (e.g., anxiety and depression). In particular, given that the long-term course of the disease is characterized by progressive deterioration [47], young patients might be at increased risk for psychological distress.

While comparing children and adolescents in remission with the healthy control group, we did not find differences on any of the HRQOL dimensions. This can be attributed to the fact that children in remission are by definition symptom-free [11]. A recent publication by the SWISS IBD Cohort even reported higher psychological functioning in IBD patients than in controls. The authors attributed this unexpected finding to the excellent social support in the young patients’ environment [41]. Walter et al. [48], on the basis of a careful examination of the literature, noted that older studies (1989 – 2004) reported higher depression rates; subsequent advances in treatment may be responsible for the lower levels of psychological distress observed more recently. Therefore, we suggest that screening and treatment for mental wellbeing should be implemented especially for those with increased disease activity and potentially weaker social support.

Our third hypothesis, following others [18, 49], was that physical activity levels (subjective and objective) would be lower in children and adolescents with IBD than in their healthy counterparts. While the three groups did not differ regarding subjectively assessed PA in our study, lower objectively assessed PA was indicated by the number of steps per day, especially in the IBD-AD group. Thus, the present data are in account with previous results, however based on objective measurements. Werkstetter et al. [14] also reported reduced amounts of steps per day in children and adolescents with IBD, while studies of adult patients have found that suffering from IBD tended to lead to a sedentary lifestyle [50]. An explanation might be that patients with active IBD are restricted and discouraged by unpredictable symptoms, physical restriction, inconvenience and discomfort [49]. Furthermore, we found that children and adolescence with active IBD perceived physical strain to be more vigorous as compared to IBD-RE and HC. This is reflected in their subjectively estimated greater intensity of the 6 MWT, while achieving less distance than the other groups and their extremely elevated self-reported vigorous PA levels over the last 7 days. This finding indicated a lower fitness in children and adolescents with active IBD, and was in line with findings of Ploeger et al. [13] Children with IBD exhibited impaired aerobic and anaerobic exercise capacity, compared to reference values.

However, a lower amount of PA might be an issue for the following reasons. Generally, regular low intensity exercise is beneficial in reducing distress and improving quality of life in young patients with IBD [51]. A UK survey revealed that PA made patients with IBD feel better and healthier, boosted energy, reduced IBD symptoms, provided an alternative focus and fostered feelings of normality [49]. On a physiological level, recent findings indicated that IBD patients could experience anti-inflammatory effects from the myokines released during skeletal muscle contraction while exercising, thereby inhibiting the release of protective heat shock proteins (Hsps), which help to regulate inflammation and immunity [52]. It was further asserted that PA is an anabolic stimulus, reducing inflammation and positively affecting growth factors, i.e., IGF-I [53]. Additionally, Robbins et al. [49] argued that regular PA should be undertaken in IBD to help maintain bone mineral density and prevent osteoporosis. We should note, however, that exaggerated amounts of (high-intensity) PA might lead to an increase in symptoms. Vigorous PA such as distance running and endurance exercise commonly might cause gastrointestinal discomfort including nausea, heartburn, and even gastrointestinal bleeding in patients with IBD [54]. By contrast, moderate PA seems to be useful in improving many aspects of the lives of patients with IBD. Nonetheless, a fear of symptoms exacerbation may be an indication that patients lack the impetus to start PA, and clinicians may be reluctant to prescribe PA, even though it might serve as a protective and preventive factor with respect to IBD. Collectively, we claim that regular moderate PA should receive greater attention in scientific intervention studies as adjuvant to prescribed pharmacotherapies.

Despite the novelty of the findings and the application of internationally validated and accepted questionnaires and objective measures, several limitations warrant against an overgeneralization of the present findings. First, the relatively small sample, especially the small number of children in an active disease state, created difficulties in detecting statistically significant effects. Though we also relied on effect sizes, which are not sensitive to sample size. Second, the recruitment of the sample was restricted to the German speaking part of Switzerland. Of these patients, only participants willing and able to participate were assessed. Even though we tried to cover an extensive range of factors, it was not possible to control for all confounders, such as microbial or environmental conditions, which might have influenced two or more outcomes in the same or in opposite directions. In this context, one suggestion for future studies might be the assessment of objective disease severity, fecal calprotectin as another objective measure. Otherwise we would suggest extending the cross sectional design to include lifestyle intervention studies and their supportive effect in the disease coping process. Last, we did not distinguish between patients with CD and UC; whereas for diagnostic reasons such an approach would have been easy to follow and justify, we decided to split patients into IBD-AD and IBD-RE, as suggested by recent research such as Reigada et al. [42, 55].

## Conclusion

The pattern of results suggests that effective medical treatment of IBD in children and adolescents is associated with favorable physiological parameters, psychological dimensions and PA. Psychological counselling of children and adolescents with severe IBD seem to be advised in addition to standard treatment schedules.

## Abbreviations

6 MWT: Six minute walking test
CD: Crohn’s disease
CDAI: Crohn’s disease activity index
ChilD-S: Child Depression Screener
CRP: C-reactive protein
ERS: Erythrocyte sedimentation rate
ES: Effect size
HC: Healthy control
HR: Heart rate
HRQOL: Health related quality of life
Hsps: Heat shock proteins
IBD: Inflammatory bowel diseases
IBD-AD: IBD in an active state of the disease
IBD-RE: IBD in remission
MET: Metabolic equivalent
PA: Physical activity
PIBD: Pediatric IBD
PUCA: Pediatric ulcerative colitis activity index
UC: Undefined colitis
